# Neurocognition, Insight and Medication Nonadherence in Schizophrenia: A Structural Equation Modeling Approach

**DOI:** 10.1371/journal.pone.0047655

**Published:** 2012-10-29

**Authors:** Laurent Boyer, Michel Cermolacce, Daniel Dassa, Jessica Fernandez, Mohamed Boucekine, Raphaelle Richieri, Florence Vaillant, Remy Dumas, Pascal Auquier, Christophe Lancon

**Affiliations:** 1 Aix-Marseille University, EA 3279 – Public Health, Chronic Diseases and Quality of Life - Research Unit, Marseille, France; 2 Department of Psychiatry, Sainte-Marguerite University Hospital, Marseille, France; 3 Department of Psychiatry, La Conception University Hospital, Marseille, France; University of Granada, Spain

## Abstract

**Objective:**

The aim of this study was to examine the complex relationships among neurocognition, insight and nonadherence in patients with schizophrenia.

**Methods:**

*Design:* Cross-sectional study. *Inclusion criteria*: Diagnosis of schizophrenia according to the DSM-IV-TR criteria. *Data collection*: Neurocognition was assessed using a global approach that addressed memory, attention, and executive functions; insight was analyzed using the multidimensional ‘Scale to assess Unawareness of Mental Disorder;’ and nonadherence was measured using the multidimensional ‘Medication Adherence Rating Scale.’ *Analysis:* Structural equation modeling (SEM) was applied to examine the non-straightforward relationships among the following latent variables: *neurocognition*, *‘awareness of positive symptoms’ and ‘negative symptoms’*, *‘awareness of mental disorder’ and nonadherence*.

**Results:**

One hundred and sixty-nine patients were enrolled. The final testing model showed good fit, with normed χ^2^ = 1.67, RMSEA = 0.063, CFI = 0.94, and SRMR = 0.092. The SEM revealed significant associations between (1) neurocognition and ‘awareness of symptoms,’ (2) ‘awareness of symptoms’ and ‘awareness of mental disorder’ and (3) ‘awareness of mental disorder’ and nonadherence, mainly in the ‘attitude toward taking medication’ dimension. In contrast, there were no significant links between neurocognition and nonadherence, neurocognition and ‘awareness of mental disorder,’ and ‘awareness of symptoms’ and nonadherence.

**Conclusions:**

Our findings support the hypothesis that neurocognition influences ‘awareness of symptoms,’ which must be integrated into a higher level of insight (i.e., the ‘awareness of mental disorder’) to have an impact on nonadherence. These findings have important implications for the development of effective strategies to enhance medication adherence.

## Introduction

The importance of maintenance therapy in schizophrenia has been well established, and long-term maintenance treatment with antipsychotic medication is critical for preventing relapse [1]. The risk of relapse increases almost 5-fold when antipsychotic drug therapy is discontinued [2]. Nonadherence to medication worsens symptoms and increases suicidal attempts and, consequently, emergency room visits or re-hospitalization [3]. Understanding the determinants of nonadherence in schizophrenia is thus important for developing effective relapse prevention strategies [4].

Over the last decades, numerous studies have investigated the characteristics of the patient, the patient's environment, or the treatment as determinants of nonadherence [4], [5]. Among these determinants, cognition and insight are important features [4], [6]. Cognitive impairments in attention, memory, and information processing may be related to patients' difficulty in managing medications [6], [7]. A lack of insight or the inability to understand one's illness is also commonly cited as a significant contributor to medication nonadherence [8], [9], [10]. Several etiological models have been proposed, each of which may play a role in nonadherence. In particular, lack of insight has been suggested to be a psychological defence mechanism as well as a neurocognitive and metacognitive deficit [11]. These last two models may be related: a minimal level of neurocognition may be necessary to perform complex integrative metacognitive acts, which allow for the construction of a complex narrative [12]. Metacognition refers to a range of semi-independent functions that allow a person to deploy, in a relatively simultaneous manner, a synthesised understanding of himself or herself as a being experiencing the illness and others as beings who respond to the experience of the illness. However, several studies have yielded contradictory results concerning the role of cognition [9], [13], [14], [15] and insight [16], [17], [18], raising doubts about their predictive power [19]. Several issues could be explored in more depth, thus casting new light on the relationships among neurocognition, insight and nonadherence in patients with schizophrenia. Prior studies often used confounded assessments that (1) did not include the three main composites of neurocognition (i.e., memory, attention, and executive functions) [13], [20], (2) considered insight to be a one-dimensional phenomenon, using for example the “Lack of Judgment and Insight” item on the Positive and Negative Syndrome Scale (PANSS) or only a few dimensions of the Scale to assess Unawareness of Mental Disorder (SUMD) [21], [22], and (3) did not distinguish between adherence behavior and attitudes towards medication [5], [19]. Moreover, although adherence is considered to be a dynamic and continuous behavior that is influenced by the complex interactions of many factors [23], most studies have examined the impact of each determinant in isolation [4], [5]. Thus, the exact nature of the interdependent relationships among neurocognition, insight and nonadherence and the relative contribution of each of these determinants remains unclear.

The aim of this study was to examine the complex relationships among neurocognition, insight and nonadherence in patients with schizophrenia. Neurocognition was assessed using a global approach that combines memory, attention, and executive functions; insight was analyzed from a multidimensional perspective; and nonadherence was measured as a continuous variable distinguishing adherence behavior and attitude toward medication. We used structural equation modeling (SEM), which is a useful statistical procedure, to test a theory involving non-straightforward relationships and is therefore well suited to the management of cross-sectional data for inferential purposes [24].

## Materials and Methods

### Study participants

The study prospectively evaluated all patients attending a day hospital over a period of 12 months, from January 2011 to December 2011. All patients provided written informed consent. The inclusion criteria were as follows: age over 18 years; diagnosis of schizophrenia according to the Diagnostic and Statistical Manual of Mental Disorders, 4th ed. (DSM-IV-TR) criteria [25]; and French as the native language. The exclusion criteria included: reduced capacity to consent, diagnosis other than schizophrenia on Axis I of the DSM-IV, decompensated organic disease and mental retardation. All clinical assessments are performed in routine practice in our university psychiatric center. According to the Article L1121-1, LOI n°2011-2012 du 29 décembre 2011 - art. 5, ethical approval is not needed for researches in which all actions are performed and products used routinely. This study was conducted in accordance with the Declaration of Helsinki and French Good Clinical Practices.

### Data collection

The following data were collected:

1. **Socio-demographic information.** Age, gender, and educational level.
2. **Clinical characteristics.** Type of schizophrenia according to the DSM-IV; duration of disease; and psychotic symptoms based on the Positive and Negative Syndrome Scale (PANSS), which comprises three different subscales: positive, negative and general psychopathology [26].
3. **Drug information.** Antipsychotic medications (first generation antipsychotics - FGAs, second-generation antipsychotics – SGAs).
4. **Neurocognitive assessment.** Several measures were selected based on previous research to test memory, attention, and executive functions. Memory was assessed using the California Verbal Learning Test (CVLT); attention was assessed using the D2 attention task; and executive function was tested using the Stroop color-word test for inhibition capacity, the verbal fluency test (letter and category domains) for spontaneous flexibility, the Trail Making Test A and B (TMT) for reactive flexibility, and the Wechsler Adult Intelligence Scale, Third Edition (WAIS–III arithmetic and symbol coding). The tests were administered in a standardized manner by the same senior psychologist, who has been intensively trained in test administration and who was not involved with the treatment of the individuals. The same instructions were given to the individuals prior to each trial.
5. **Insight into illness.**Was assessed using the short form of the Scale to Assess Unawareness of Mental Disease (SUMD) [27], which is a semi-structured interview designed to assess 9 items of awareness: (1) having a mental disorder, (2) need to take medication, (3) consequences, (4) hallucinations, (5) delusions, (6) thought disorder, (7) blunted affect, (8) anhedonia, and (9) asocialty. Each of these domains was rated on a 4-point rating scale: 0 - not applicable, 1 – aware, 2 - somewhat aware/unaware, and 3 - severely unaware. The shortened version of the SUMD describes 3 dimensions: awareness of the mental disorder (items 1–3), level of awareness of positive symptoms (items 4–6) and level of awareness of negative symptoms (items 5–9) [28], [29], [30]. Scores for each dimension were obtained by summing the items within each dimension. Dimension scores ranged from 0, indicating the highest insight, to 9, the lowest insight.
6. **Nonadherence.** Was assessed with the Medication Adherence Rating Scale (MARS) [31]. It is a 10-item yes/no (1/0) self-reporting multidimensional instrument describing 3 dimensions: ‘medication adherence behavior’ (items 1–4), ‘attitude toward taking medication’ (items 5–8) and ‘negative side-effects and attitudes to psychotropic medication’ (items 9 and 10). Scores for each dimension are obtained by summing the items within each dimension. A low score is correlated with a low likelihood of medication adherence, and a high score is correlated with a high likelihood.

### Hypotheses

To apply structural equation modeling, we constructed hypothetical relationships among the variables by examining previously published research. We hypothesized that neurocognition influences nonadherence, both directly [6] and indirectly via insight [20]. We proposed that insight mediates the relationship between neurocognition and nonadherence. We hypothesized that neurocognition mainly influences the dimensions ‘awareness of positive symptoms’ and ‘negative symptoms’ of the SUMD and affects the dimension ‘awareness of mental disorder’ to a lesser extent [32], [33]. We also suggest that ‘awareness of positive symptoms’ and ‘negative symptoms’ influence (e.g., is integrated into) the dimension ‘awareness of mental disorder’ based on the following definition of insight [34]: an element of a larger personal and interpersonal understanding of one's illness, based on a self-representation that is more complete than simply possessing a piece of knowledge on the illness (e.g., on symptoms). Furthermore, we proposed that, among the insight dimensions, ‘awareness of mental disorder’ would constitute the main determinant of nonadherence [35], [36], especially the ‘attitudes towards medication’ dimension [10], [19], [23], [37].

**Figure 1:**
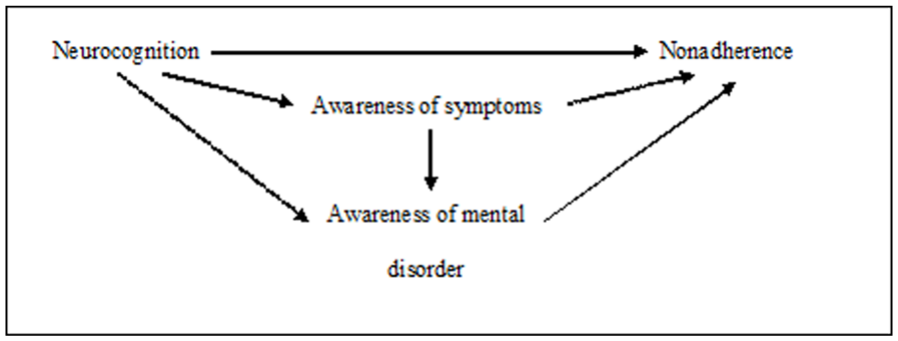
Hypothetical sequential process.

The models tested were thus based on the following hypothetical sequential process (Figure 1).

### Statistical analysis

Data analysis was conducted in two phases.

Correlational analyses among neurocognition, insight and nonadherence were performed using Pearson correlation coefficients. This statistical analysis was performed using the SPSS version 17.0 software package (SPSS Inc., Chicago, IL, USA). All tests were two-sided, and statistical significance was defined as p<0.0005 to correct for multiple hypothesis testing.

SEM was then conducted with LISREL 8.52 for Windows. Our model was based on several latent variables, namely neurocognition, awareness of positive symptoms and negative symptoms, awareness of mental disorder, and nonadherence. We evaluated model fit using the chi-squared statistic, root mean square error of approximation (RMSEA), the comparative fit index (CFI), and the standardized root mean square residuals (SRMRs) [38]. A small (less than 3), nonsignificant chi-squared value indicates that the observed correlations are not significantly different than the expected correlations. The RMSEA indicates how well the model would fit the hypothetical population covariance matrix. A value less than 0.05 is indicative of a close-fitting model, between 0.05 and 0.08 is indicative of a reasonable fit, and 0.10 or greater indicates a poor model. The CFI indicates the extent to which the hypothesized model provides a better fit than the null model. The comparative fit index (CFI) has a range of 0–1.0; a value greater than 0.90 suggests a reasonably good fit. Finally, SRMR (i.e., the average difference between the correlations predicted by the model and the observed correlations) values less than 0.10 indicate good fit. The path coefficients, which can range from −1 to +1, indicate the strength and sign of the paths. The significance of the path coefficient is assessed using the standard errors and the t-values for each coefficient. In addition to the statistical significance of the path coefficients, the strength of the relationship plays a role in determining whether the relationships are weak (<0.2), moderate (0.2–0.5) or strong (>0.5) [39].

## Results

### Sample characteristics

One hundred and sixty-nine outpatients with schizophrenia were enrolled in our study. The mean age was 36.6 years (±12.5 years), 73.4% (n = 124) were male, 49.7% (n = 84) had a university level education, and 63.4% (n = 111) suffered from paranoid schizophrenia. The mean duration of illness was 12.5 years (±10.0 years). The patients show a moderate severity of symptoms with a total PANSS score of 58.5 (±16.0); the subscores were 14.4±5.4, 19.7±7.3, and 36.2±10.1, respectively, for the positive, negative, and general psychopathology factors, and 86.7% had been taking second-generation antipsychotics. The mean neurocognition, SUMD and MARS scores are presented in Table 1.

**Table 1:** Intercorrelations of cognitive, insight, and nonadherence variables.

	Mean	SD	1	2	3	4	5	6	7	8	9	10	11	12	13	14	15
1. Medication adherence behavior	2.6	1.2	1.000														
2. Attitude toward taking medications	2.7	1.1	**.265** **	1.000													
3. Negative side-effects and attitudes toward psychotropic medications	1.1	0.8	.260	**.310** **	1.000												
4. D2 attention task	350.6	110.2	−.262	−.087	−.084	1.000											
5. TMT-A (Time)	44.7	24.8	.049	−.027	−.112	**−.513** **	1.000										
6. TMT-B (Time)	112.6	67.4	.078	.084	−.103	**−.427** **	**.676** **	1.000									
7. Stroop interference	37.0	13.3	−.141	.124	.059	**.508** **	**−.508** **	**−.562** **	1.000								
8. Letter fluency	18.6	7.2	−.020	.083	.094	.235	**−.373** **	−.217	.320	1.000							
9. Category fluency	24.2	7.9	−.005	.076	.205	.242	**−.375** **	**−.368** **	**.380** **	**.499** **	1.000						
10. CVLT List A - 1–5	42.9	12.5	−.010	.037	.079	**.357** **	**−.424** **	**−.363** **	**.366** **	**.321** **	**.378** **	1.000					
11. WAIS III - Digit Symbol-Coding	6.0	3.2	−.055	−.047	.071	**.504** **	**−.469** **	**−.407** **	**.490** **	**.386** **	**.417** **	**.422** **	1.000				
12. WAIS III - Arithmetic	7.0	3.2	−.192	−.082	.025	**.442** **	**−.335** **	**−.444** **	**.508** **	**.316** **	**.357** **	**.321** **	**.540** **	1.000			
13. Awareness of mental disorder	4.4	1.9	−.084	**−.315** **	−.040	.110	−.015	.032	.024	−.066	−.074	−.092	.006	.100	1.000		
14. Awareness of positive symptoms	4.2	2.3	−.018	−.041	−.136	−.134	.080	.295	−.176	−.172	**−.361** **	−.220	−.186	−231	**.465** **	1.000	
15. Awareness of negative symptoms	4.0	2.1	−.132	−.077	−.122	.079	.006	.114	−.051	−.202	−.301	**−.328** **	−.173	−.130	**.311** **	**.432** **	1.000

** p≤0.0005.

### Correlations among variables

**Figure 2:**
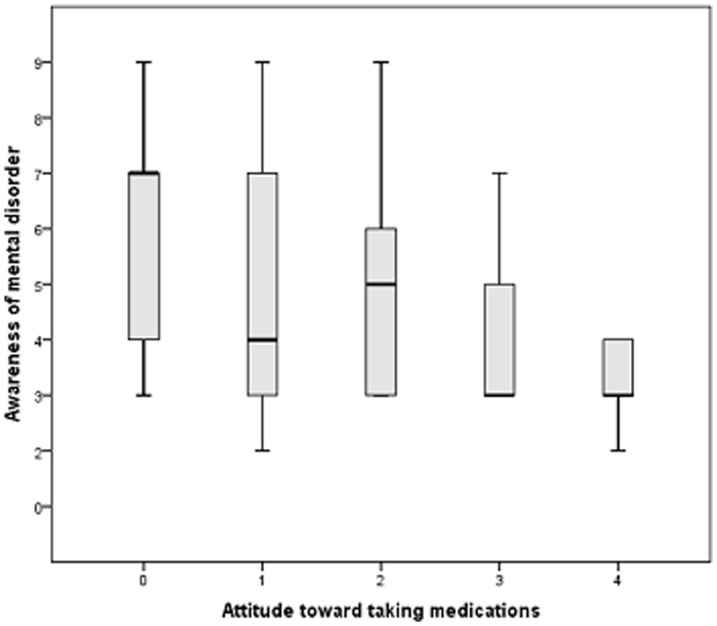
Boxplot presenting the distribution of the ‘awareness of the mental disorder’ dimension score according to the level of ‘attitude towards taking medication.’

The correlations are provided in Table 1. Neurocognitive capacities and MARS scores were not significantly correlated. On the contrary, neurocognition was associated with insight but only for the ‘positive and negative symptoms’ dimensions. Two significant correlations were found with the CVLT, and category fluency tests (respectively r = −0.33 and r = −0.36). ‘Awareness of mental disorder’ was the only dimension that was significantly associated with the ‘attitude toward taking medication’ dimension of the MARS (r = −0.32). In figure 2, a boxplot presents the distribution of the ‘awareness of the mental disorder’ dimension score according to the level of the ‘attitude towards taking medication.

### Structural equation model

**Figure 3:**
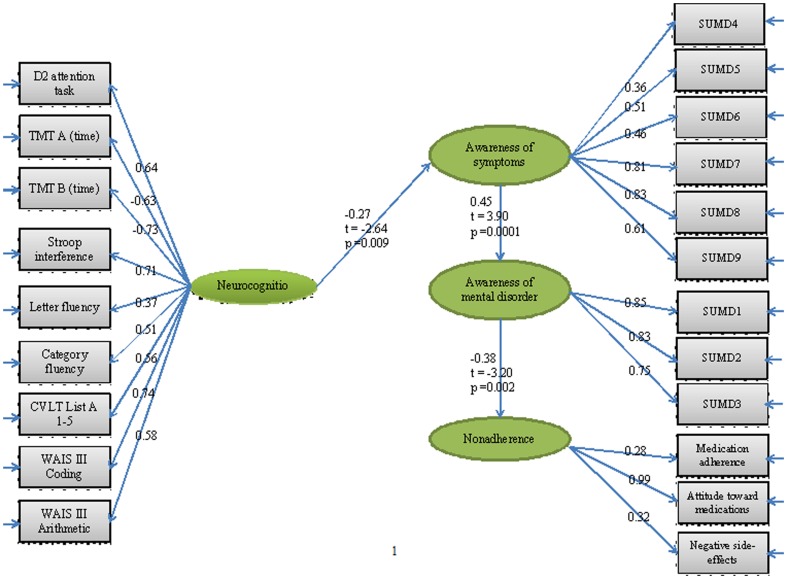
Hypothesized model with fitted coefficients.

The structural equation model fitted to assess the hypothesized model is illustrated in Figure 3. We began with a theoretical path model based on our hypotheses. Three paths were not significant, including neurocognition – nonadherence (T-value = −1.31), neurocognition – ‘awareness of mental disorder’ (T-value = 1.88) and ‘awareness of symptoms’ - nonadherence (T-value = 0.57); thus, they were removed. The final testing model showed good fit based on the chi-squared statistic (normed χ^2^ = 1.67) and had RMSEA = 0.063, CFI = 0.94, and SRMR = 0.092. The SEM revealed significant but moderate associations between (1) neurocognition and ‘awareness of symptoms’ (path coefficient = −0.27), (2) ‘awareness of symptoms’ and ‘awareness of mental disorder’ (path coefficient = 0.45), and (3) ‘awareness of mental disorder’ and nonadherence (path coefficient = −0.38), mainly in the ‘attitude toward taking medication’ dimension (path coefficient = 0.99).

## Discussion

This study investigated, through structural equation modeling, the influence of neurocognition and insight on nonadherence among patients suffering from schizophrenia. Our results highlight the non-straightforward relationships among neurocognition, insight, and nonadherence. Multidimensional assessments of neurocognition, insight and nonadherence combined with SEM allowed us to develop path model that accounted for the complexity of the relationships and may explain some contradictory results from previous studies. In particular, our multidimensional analysis of insight shows different links between the dimensions of insight and neurocognition or nonadherence, which supports the differences found in previous studies [8], [9], [10], [16], [18].

Our findings better explain the mechanisms for nonadherence in patients with schizophrenia. Not all the facets of insight were associated with neurocognition and nonadherence, suggesting that a multistep approach to address insight formation is necessary to improve nonadherence. The direct influence of neurocognition on ‘awareness of symptoms,’ which is particularly strong for negative symptoms (all path coefficients >0.6), is in accordance with several previous studies [32], [33], confirming that insight can be considered as a failure of competence to recognize the symptoms and illness (neurocognitive model) [40]. The unawareness of positive symptoms (path coefficients ≤0.5) appeared less associated with neurocognition than negative symptoms, suggesting a mechanism other than neurocognitive deficit. Interestingly, Mohamed et al. [33] suggested that unawareness of positive symptoms may also reflect an attempt to avoid the negative connotations of the illness. However, neither neurocognition nor ‘awareness of symptoms’ alone influenced medication nonadherence, suggesting that ‘awareness of symptoms’ must be integrated into a higher level of insight (i.e., the ‘awareness of mental disorder’) to affect nonadherence. Interestingly, several studies have reported that awareness of a mental disorder, including having a mental disorder, the need to take medication, and the consequences of the mental disorder may result from metacognitive capacities, in particular theory of mind (ToM) or mentalizing (i.e., the ability to attribute mental states to one's self and others) [30], [41], [42]. This metacognitive model propose a ‘social’ aspect of insight relied upon the ability of the patient to accept and incorporate others' opinion (e.g., professionals, friends, family) about psychosis into one's own insight. We thus hypothesize that neurocognitive and metacognitive conceptions of insight may represent a continuous process necessary to have an impact on nonadherence [43], in which patients with schizophrenia gain knowledge about the illness (awareness of symptoms) and then subsume their ‘awareness of symptoms’ into the ‘awareness of having mental disorder’ by accepting the other perspectives in a context of social situations. ‘Unawareness of positive symptoms’ and a possible psychological defense may be involved in this continuous process, which leads to nonadherence but to a lesser extent than neurocognition and metacognition.

The relationships among neurocognition, insight and nonadherence should be considered to develop an effective strategy to enhance medication adherence. Our findings support the development of complementary therapeutic approaches, such as cognitive remediation combined with psycho-social rehabilitation. Cognitive remediation may ameliorate deficits in cognitive functioning, which may in turn limit the clinical benefits derived from psychoeducation [44]. A recent meta-analysis has revealed that psychoeducation alone failed to have any influence on adherence [16]. However, there is a need to propose psycho-social rehabilitation, especially functional skills training [45]. Targeted social cognitive intervention leads to improvements in social cognition [46], which may specifically enhance the quality of the relationship between the patient and the caregivers and the therapeutic alliance between the patient and the professional, which are important determinants of nonadherence [8], [47]. In addition, our findings support interest in newly developing forms of individual psychotherapy that offer individuals with schizophrenia opportunities to develop metacognitive capacity and to construct more complex narratives of their lives and challenges [48], [49]. Finally, the relationship between insight and attitudes toward taking medication confirmed previous studies [10], [19], [23], [37] and suggest that clinicians should detect problematic medication attitudes on an individual basis. Modification of attitudes should be targeted by professionals or during psychoeducation programs as an indirect way of enhancing medication adherence [19].

The impact of awareness of the mental disorder on nonadherence remains moderate in strength. One explanation is that reasons for nonadherence are multifactorial [4], [5], and multiple approaches, beyond the problem of insight, are necessary to address the complex problem of nonadherence. However, we also wonder about the limits of the concept of insight as a predictor of nonadherence. This weak relation was already found in previous studies [19], which identified the importance of patients' individual point of view, experience, and health beliefs in complement to ‘objective’ determinants such as insight.

### Limitations and perspectives

There are several limitations to this study.

Adherence behavior is not easy to detect and quantify, and all methods of detection have some drawbacks. As such, the use of the MARS may be criticized. This scale is a subjective method of assessing adherence in comparison with objective methods such as pill counts, pharmacy records, electronic monitor and plasma concentrations. However, as suggested by Velligan et al., even the use of more objective measures can be associated with significant errors [5]. Moreover, the MARS has several advantages and qualities. It reflects an understanding that adherence is a continuous variable, has good psychometric properties, predict satisfactorily nonadherence and is widely used.

Our study concerned only one insight instrument, which is a researcher-rated method of assessment. Although the SUMD is valid and reliable for assessing insight [27], previous studies have also established that a moderate correlation exists between researcher-rated and self-report insight scales because each instrument may measure a different aspect of insight [50]. It would be interesting to determine whether our findings can be replicated with other insight instruments.

This study is also limited by the fact that it is cross-sectional rather than prospective in design. No causal inference can be formally advanced, and our model should be interpreted from an associational point of view. However, we based our modeling on plausible hypotheses based on previous studies. Future studies are needed to establish whether the model reported herein is longitudinally robust and to confirm that the sequences tested in the model are temporally verified.

Considering the study sample, patients were mostly middle-aged males with mild disease severity and more than 5 years of illness duration. Confirmation is therefore required on more diverse and larger groups of patients.

Finally, our findings should be confirmed in future studies by specifically assessing metacognitive capacities and testing the links with neurocognition, insight and nonadherence.

## Conclusion

In conclusion, our findings suggest a continuous process that combines the cognitive and metacognitive capacities into the insight formation necessary to have an impact on nonadherence. After replication with longitudinal approaches, these findings may support complementary therapeutic approaches, such as cognitive remediation combined with psycho-social rehabilitation, to enhance both insight and medication adherence.
